# Regulation of NKT Cell Localization in Homeostasis and Infection

**DOI:** 10.3389/fimmu.2015.00255

**Published:** 2015-05-27

**Authors:** Drew Slauenwhite, Brent Johnston

**Affiliations:** ^1^Department of Microbiology and Immunology, Dalhousie University, Halifax, NS, Canada; ^2^Department of Pediatrics, Dalhousie University, Halifax, NS, Canada; ^3^Department of Pathology, Dalhousie University, Halifax, NS, Canada; ^4^Beatrice Hunter Cancer Research Institute, Halifax, NS, Canada

**Keywords:** natural killer T cells, chemokines, cytokines, homeostasis, leukocyte homing

## Abstract

Natural killer T (NKT) cells are a specialized subset of T lymphocytes that regulate immune responses in the context of autoimmunity, cancer, and microbial infection. Lipid antigens derived from bacteria, parasites, and fungi can be presented by CD1d molecules and recognized by the canonical T cell receptors on NKT cells. Alternatively, NKT cells can be activated through recognition of self-lipids and/or pro-inflammatory cytokines generated during infection. Unlike conventional T cells, only a small subset of NKT cells traffic through the lymph nodes under homeostatic conditions, with the largest NKT cell populations localizing to the liver, lungs, spleen, and bone marrow. This is thought to be mediated by differences in chemokine receptor expression profiles. However, the impact of infection on the tissue localization and function of NKT remains largely unstudied. This review focuses on the mechanisms mediating the establishment of peripheral NKT cell populations during homeostasis and how tissue localization of NKT cells is affected during infection.

## Introduction

The clearance of bacterial, viral, fungal, and protozoan infections depends upon the coordinated activation of both the innate and adaptive arms of the immune system (1). Innate immune cells, such as macrophages, dendritic cells (DCs), neutrophils, and natural killer (NK) cells, are critically involved in the initial control and clearance of infectious organisms. However, adaptive immunity mediated by T cells and B cells is also required to generate specific sterilizing responses and provide long-lasting immunological memory. Natural killer T (NKT) cells are a subset of T cells that serve as a bridge between innate and adaptive immunity. Upon activation, NKT cells rapidly generate and secrete a diverse array of cytokines and chemokines (2–4), allowing them to shape the magnitude and polarization of host immune responses in infection (5, 6), autoimmune disease (7, 8), allergy (9, 10), and cancer (11). Compared to conventional T cells, NKT cells exhibit altered patterns of tissue localization, suggesting differences in the signals regulating homing and homeostasis. This review examines pathways important in the trafficking and maintenance of NKT cell populations under homeostatic conditions and during microbial infections. The impact of these mechanisms on NKT cell-derived anti-microbial effector functions will be discussed in terms of their ability to orchestrate both innate and adaptive immune responses.

## Natural Killer T Cells

Natural killer T cells develop in the thymus from uncommitted thymic progenitors that undergo T cell receptor (TCR) rearrangement and selection. However, unlike the diverse TCR repertoire of conventional T cells that are selected via type I or type II major histocompatibility complex (MHC), NKT cells express a restricted repertoire of TCR rearrangements that are selected via the MHC-like molecule CD1d (12, 13). While the specific selecting antigen(s) in the thymus remains unclear, several endogenous NKT cell ligands have been proposed based on NKT cell activating capacity. These include isoglobotrihexosylceramide (iGb3) (14), lysophosphatidylcholine (LPC) and lysosphingomyelin (LSM) (15), the peroxisome-derived ether-bonded compounds lysophosphatidylethanolamine (pLPE) and lysophosphatidic acid (eLPA) (16), β-glucosylceramide (β-GluCer) (17), and α-glycosylceramides (18). However, the relative roles of these candidate ligands during intrathymic NKT cell development in mouse versus human as well as their capacities to influence NKT cell functional regulation and/or tissue localization in the periphery remain undefined.

Isoglobotrihexosylceramide, which appeared to be a promising candidate for an endogenous NKT cell selecting antigen in mice, is unlikely to be an endogenous ligand for human NKT cells due to the lack of the relevant iGb3 synthase enzymes in humans (19). Furthermore, the contamination of commercial β-GluCer with α-linked species has brought into question the role of this compound. Indeed, two groups have demonstrated that highly purified preparations of β-GluCer lack NKT cell stimulatory activity (18, 20). However, Kain and colleagues (18) identified the presence of small quantities of endogenous α-linked glycosylceramides [α-GluCer and α-galactosylceramide (α-GalCer)], a class of glycolipids that were thought to be absent in mammalian cells, and identified them as possible endogenous ligands for NKT cell selection and activation. Our understanding of NKT cell development and function will continue to improve as ongoing efforts further characterize the self-lipid antigens that select NKT cells in the thymus.

Two major subsets of NKT cells can be distinguished based on their TCR repertoire and lipid reactivity. Type I or invariant NKT (*i*NKT) cells express an invariant TCRα chain composed of Vα14–Jα18 rearrangements in mice and Vα24–Jα18 in humans, paired with a restricted repertoire of Vβ chains (Vβ8.2, Vβ7, or Vβ2 in mice, and Vβ11 in humans) (21, 22). Specific detection of *i*NKT cells is possible through the use of CD1d tetramers loaded with α-GalCer (23, 24). Analogs of α-GalCer are potent activators of *i*NKT cells and can influence immune responses in many pathological states, including microbial infection (25, 26), autoimmune disease (7), allergy (27), and cancer (28). Type II NKT cells are CD1d-restricted but do not recognize α-GalCer (29). This is a more heterogeneous population of cells, expressing oligoclonal TCRs that utilize a limited collection of Vα (Vα1, Vα3, Vα8) and Vβ-rearrangements (29–31). Comparisons of Jα18^−/−^ mice lacking type I NKT cells with CD1d^−/−^ mice lacking type I and type II NKT cells suggest that the type II NKT cells are regulatory cells that can suppress anti-tumor immunity (32–34). The best characterized subset of type II NKT cells expresses a TCR that recognizes sulfatide (3-sulfated galactosylceramide) (29, 31, 35). These cells serve as an important regulatory population during inflammatory responses and can be activated by sulfatide to suppress autoimmunity (36–40). Type II NKT cells can also regulate *i*NKT cell responses. For example, activation of type II NKT cells by sulfatide suppresses the proliferative and cytokine responses of *i*NKT cells activated with α-GalCer (33). Furthermore, in a ConA-induced hepatic injury model, sulfatide-activated type II NKT cells induced *i*NKT cell anergy and prevented inflammatory liver disease (37). However, dysregulated responses of type II NKT cells have also been shown to play a role in the pathogenesis of inflammatory bowel disease in both mice and humans (41, 42). While *i*NKT cells are more prevalent than type II NKT cells in mice, type II NKT cells appear to be the predominant subset in humans (43). This review focuses on responses of *i*NKT cells, and the term NKT cell will be used throughout to refer to this population.

In addition to TCR–CD1d interactions, NKT cells are also stimulated by inflammatory cytokines (44–46), neurotransmitters (47), and toll-like receptor (TLR) ligands (48–50). Following activation, NKT cells are able to produce a wide range of cytokines including interferon-γ (IFN-γ), tumor necrosis factor (TNF), interleukin-2 (IL-2), IL-4, IL-10, IL-13, IL-17, IL-21, IL-22, and granulocyte-macrophage colony-stimulating factor (GM-CSF) (2–4, 51). However, the cytokine profile is influenced by the nature of the stimulation and the subset of NKT cells that are activated. Indeed, recent studies have identified a number of distinct lineages of NKT cells that emerge during development, each with a unique profile of transcription factors and cytokine production (52–59). Based on these profiles, NKT cells can be subdivided into NKT-1, NKT-2, NKT-17, and NKT-10 subsets, analogous to the T helper type 1 (Th1), Th2, Th17, and IL-10 producing subsets of conventional T cells. Through their secretion of various cytokines, NKT cells are able to activate other immune cells, contributing to NK cell transactivation (60), DC maturation (61, 62), T cell polarization (63, 64), and B cell antibody responses (65).

## NKT Cell Homeostasis

Natural killer T cells require a number of growth factors and survival signals for their maintenance in the periphery. In contrast to the requirement for CD1d during thymic NKT cell selection and initial maturation, mature NKT cells do not require continual CD1d interactions in the periphery to support homeostatic proliferation, long-term survival, or to maintain tissue distribution (66). Instead, NKT cells rely more on signaling elicited by cytokines such as IL-15, and to a lesser extent IL-7 (66–69). However, while thymic NKT cell development and homeostatic NKT cell proliferation are impaired in IL-15-deficient mice, these populations are not abolished (67, 70). It is possible that this is due to differences in the requirement for IL-15 during development of distinct NKT cell lineages. For example, NKT-1 cells express CD122 (the IL-2/IL-15 receptor β-chain) and require IL-15 for development and homeostasis, while NKT-2/NKT-17 cells (marked by the expression of IL-17RB, a receptor for IL-25) develop normally in the absence of IL-15 (58). Accordingly, CD122 is moderately to highly expressed on NKT cells in the mouse liver and spleen, where the NKT-1 lineage constitutes the majority of the NKT population, but is not expressed on lymph node NKT cells, where NKT-17 cells are enriched (52, 54). ICOS/ICOSL interactions are also required for NKT cell homeostasis and function as survival of wild-type NKT cells transferred into ICOSL^−/−^ mice was reduced, and ICOS^−/−^ NKT cells were impaired in their ability to produce IL-4 and IL-13 (71). Many studies have highlighted roles for a variety of signaling molecules and transcription factors in NKT cell development and homeostasis, including NF-κB (72), T-bet (73, 74), c-Myc (75, 76), mTORC2 (77), calcineurin (78), Egr-2 (78), Id2 (79), Bcl-2 (80), Bcl-X_L_ (81), in addition to cytokine receptor subunits IL-2Rβ (82), IL-7Rα (83), IL-15Rα (84, 85), and the common gamma chain (83).

In addition to these factors, chemokine receptor signaling has also been implicated in regulating NKT cell homeostasis in the periphery. NKT cells express high levels of CXC chemokine receptor 6 (CXCR6) (86–89), and NKT cells in the liver and lungs are depleted in mice lacking CXCR6 or its ligand CXCL16 (90–92). Geissmann et al. (90) reported that NKT cells from CXCR6^−/−^ mice underwent apoptosis more rapidly in culture than NKT cells from CXCR6^−/+^ mice. *In vivo* however, CXCR6^−/−^ and CXCR6^−/+^ mice exhibited a similar frequency of apoptotic CD1d-reactive cells in liver sections and freshly isolated liver lymphocytes (90). We found no difference in the apoptosis rates of cultured NKT cells purified from the livers of CXCR6^+/+^ and CXCR6^−/−^ mice (91), but observed an accumulation of NKT cells in the bone marrow, suggesting an alteration in homing. Interestingly, mice deficient in Id2 exhibit impaired survival of liver NKT cells, which is associated with reduced expression of CXCR6 and the survival factors Bcl-2 and Bcl-X_L_ (79). Similarly, hepatic NKT cells from CXCR6-deficient mice expressed lower levels of Bcl-2, suggesting a role in survival (79). Despite the conflicting reports, it seems likely that CXCR6 plays a role in regulating survival of NKT cells within certain tissue environments [since NKT cell numbers are normal in most tissues (90–92)], or under specific culture conditions.

A separate study found that NKT cells in CC chemokine receptor 5 (CCR5)-deficient mice were resistant to activation-induced apoptosis, and produced more IL-4, resulting in enhanced liver injury in a model of ConA-induced hepatitis (93). Interestingly, despite an impairment of activation-induced cell death, there were no defects in Fas-mediated apoptosis in these NKT cells. In human T cells, CCR5-dependent apoptosis has been reported in response to high concentrations of the chemokine ligand CCL5 (94), or ligation of CCR5 by the human immunodeficiency virus (HIV) envelope protein gp160 (95). In these cases however, there was enhanced susceptibility to caspase-8-dependent cell death through induction of FasL (95). These studies point to a role for chemokine receptors in influencing lymphocyte survival and add to a growing body of literature demonstrating the ability of chemokine receptors to regulate a number of cellular functions in addition to their traditional roles in regulating leukocyte recruitment and positioning.

Natural killer T cell homeostasis is also regulated by the microbiome. Germ-free Swiss-Webster and C57BL/6 mice exhibit variable alterations in thymic, spleen, and liver NKT cell populations compared to conventionally housed animals (96–98). This variability may reflect differences in the conventional microbiota in control mice housed in different facilities (98). However, germ-free mice consistently exhibited increased numbers of NKT cells in the intestinal lamina propria and lungs (96, 98). NKT cell accumulation appears to result from dysregulated CXCL16 expression, and could be reversed by CXCL16 blockade or neonatal exposure to conventional microbiota (96). Bacteria of the genera *Bacteroides* comprise >50% of the bacteria in the human gut (99), and *B. fragilis* has been shown to generate α-GalCer derivatives capable of regulating NKT cells (100, 101). One such compound, α-GalCer_Bf_, binds to CD1d and activates NKT cells *in vitro* and *in vivo*, albeit to a lesser degree than synthetic α-GalCer (100). However, colonization of germ-free mice with *B. fragilis* led to variable expansion of NKT cells (100). *B. fragilis* also generates GSL-Bf717, an α-GalCer analog that inhibits NKT cell activity and restored NKT cell homeostasis in germ-free mice (101). Therefore, it appears that the composition of the intestinal microbiota influences the homeostasis of NKT cells within the colon and lungs, and may also exert influences on NKT cells within other tissues. Adding further complexity, NKT cells also influence bacterial colonization in the intestine (102), and engagement of epithelial CD1d contributes to intestinal epithelial cell-dependent regulation of mucosal homeostasis via IL-10 production (103), highlighting the intricate interactions which take place between host cells and the microbiota.

## NKT Cell Tissue Localization Patterns

In mice, NKT cells are first detected in the thymus at day 5–6 after birth, and in the periphery after day 8 (12, 104). They populate multiple tissues and reach steady state levels by 5–6 weeks of age. In the adult mouse, NKT cell frequency is highest in the liver (12–30% of liver lymphocytes), with lower frequencies in the spleen (1–3%), lungs (5–10%), thymus (0.5–1%), bone marrow (0.4–8%), lymph nodes (0.2–1%), intestines (0.05–0.6%), and blood (0.2%) (23, 24, 98, 105–110).

In contrast to the post-natal NKT cell ontogeny in mice, NKT cells are detected in the human fetal thymus at the start of the second trimester, but the frequency declines with gestational age to reach low levels in the post-natal thymus (111, 112). Human NKT cells also distribute to the periphery during the second trimester, with a prominent distribution to the small intestine which may act as a maturation site (113). Overall, the tissue distribution of NKT cells in the periphery appears to be similar between adult humans and mice. However, the frequency of NKT cells in humans is significantly lower and is subject to considerable variability among individuals. For example, frequencies of NKT cells range from 0.05 to 1% of liver lymphocytes in humans (114, 115), and generally account for 0.01–0.1% of human peripheral blood mononuclear cells, but have been observed to constitute upwards of 3% of peripheral blood mononuclear cells in some individuals (112, 116–118). The variability in NKT cell frequency between individuals appears to be influenced by genetic factors as evidenced by identical twin studies (118). Despite these differences, NKT cells play important roles in human health and disease. Indeed, dysfunctional NKT cell responses and reduced circulating numbers of NKT cells have been reported in patients with autoimmune disorders (8) and malignancies (119–122), suggesting a role for NKT cells in maintaining immune homeostasis.

## Phenotypic and Functional Differences in NKT Cell Subsets

Even though NKT cells have a restricted TCR profile, they contain phenotypically and functionally diverse subpopulations characterized by differences in surface marker expression, tissue localization, and effector functions. NKT cells in humans can be divided into CD4^+^ (12–36%), CD4^−^CD8^−^ (DN; 60–85%), or CD4^−^CD8α^+^ (1–5%) subsets (123). While the DN and CD8α^+^ subsets in human blood are phenotypically and functionally similar, the CD4^+^ subset represents a functionally distinct lineage with marked differences in cytokine profile and homing receptor expression (see Table 1) (4, 87, 116). For example, CD4^−^ NKT cells produce primarily Th1 cytokines such as IFN-γ and TNF, while CD4^+^ NKT cells generate both Th1 and Th2 cytokines (IFN-γ, TNF, IL-4, IL-5, IL-10, and IL-13) (4, 87, 116). However, tissue-resident NKT cells may have differences in surface marker expression and cytokine profiles (115, 124).

**Table 1:** **Chemokine receptor expression and ligand responses on circulating human NKT cells**.

Receptor	Expression (%)^a^			Chemotactic response^c^	Reference
	CD4^+^	DN^b^	CD8^+^	
CCR1^d^	2–25	55–85	30–80	CD4^+^; DN^++^; CD8^++^	(87, 89)
CCR2^d^	60–80	95–99	65–99	+++	(87, 89)
CCR3	0–4	0–4	0–4	n.d.	(87, 89)
CCR4	12–40	4–18	2–10	CD4^++^; DN^+^; CD8^−^	(87, 89)
CCR5	45–80	90–99	70–99	+	(4, 87, 89, 116)
CCR6	10–68	72–95	50–85	CD4^+^; DN^+++^; CD8^+++^	(87, 89, 116)
CCR7	11–28	7–32	2–25	++	(4, 87, 89)
CCR8	11–55% total NKT cells			−	(89, 140)
CCR9	0–4	0–4	0–4	−	(87, 89)
CCR10^e^	n.d.	n.d.	n.d.	−	(89)
CXCR1	5–10	3–8	n.d.	−	(89)
CXCR2	0–2	0–1	n.d.	−	(89)
CXCR3	75–90	95–99	80–90	+++	(4, 87, 89)
CXCR4	90–99	98–99	95–99	+++	(4, 87, 89)
CXCR5	0–4	0–4	0–4	−	(87, 89)
CXCR6	22–45	85–99	60–98	++	(87, 89, 116)
CX_3_CR1	4–12	4–12	n.d.	−	(89)

*^a^Range in reported frequency of receptor positive NKT cells*.

*^b^DN = CD4^−^CD8^−^double negative NKT cells*.

*^c^Net chemotactic migration: − (did not respond), + (2–10%), ++ (11–30%), and +++ (>30%)*.

*^d^Others report CCR1 and CCR2 on <2% of NKT cells (89, 116)*.

*^e^CCR8 and CCR10 mRNA detected in NKT cell subsets (89)*.

*n.d. – not determined*.

Natural killer T cells in mice are comprised of CD4^+^ (60–80%, depending on the tissue) and DN subsets, while CD8^+^ CD1d-restricted NKT cells are absent (24, 105, 125). A Th2-like subset of CD4^+^ NKT cells localizes to the lungs and contributes to airway hyperreactivity and asthma (126, 127), while a subset of DN IL-17-producing NKT cells localize preferentially to the lymph nodes and skin (54). Although there is little evidence for differences in cytokine profiles of CD4^+^ and DN NKT cell subsets in the liver and spleen, functional differences have been reported. For example, DN NKT cells from the liver are able to control tumors better than CD4^+^ NKT cells from the liver or NKT cells from other tissues (128). NKT-1, NKT-2, NKT-10, NKT-17, and follicular helper-type NKT (NKT_FH_) subsets of NKT cells have recently been identified on the basis of transcription factor profiles and select surface marker expression (52–59, 129). However, more work is needed to determine the maintenance and plasticity of these profiles since the population ratios in mice seem to change significantly over time (55). Although NKT-1, NKT-2, and NKT-17 subsets emerge in the thymus, it is likely that tissue-specific factors and microenvironmental influences act to shape the phenotype and function of NKT cells after recruitment to specific tissue sites.

## Expression of Homing Receptors on NKT Cells

The trafficking behaviors of naïve and effector/memory lymphocyte subsets are a function of the specific combinations of adhesion molecules and chemokine receptors that they express. For example, naïve T lymphocytes use l-selectin (CD62L), the αLβ2-integrin (CD11a/CD18; LFA-1), and CCR7 to enter peripheral lymph nodes at high endothelial venules (130–132), while gut homing memory lymphocytes express the α4β7-integrin and CCR9 (133, 134), and memory lymphocytes targeted to the skin express the cutaneous lymphocyte antigen (CLA) and CCR4 (135). Cells capable of migrating to sites of inflammation display varying levels of CCR1, CCR2, CCR5, CXCR3, or other chemokine receptors on their surface (130, 136, 137).

### Human NKT cells

Generally, human NKT cells express homing receptors for extra-lymphoid tissues (Table 1), with only 10–20% of circulating NKT cells expressing the lymph node homing receptor CCR7 (87, 89). Few blood NKT cells express the chemokine receptors CCR3, CCR9, CXCR1, CXCR2, CXCR5, or CX_3_CR1 (87, 89). In contrast, a majority (>60%) of NKT cells express CCR2, CCR5, CXCR3, and CXCR4, with differential expression of CCR1, CCR4, CCR6, and CXCR6 depending on the specific NKT cell subset or their tissue distribution (87, 88, 138). Multiple studies examining chemokine receptor expression on human NKT cells have observed greater frequencies of DN and CD8^+^ NKT cells expressing CCR1, CCR2, CCR5, CCR6, CXCR6, and the integrin CD49a, while CCR4 was expressed by a greater proportion of CD4^+^ NKT cells (4, 87, 89, 116, 138). The frequency of circulating NKT cells that express CCR8, a skin homing receptor expressed on the majority of human T cells in healthy skin (139), ranges from 11 to 55% (140). Adhesion molecules such as CLA, CD62L, and α4β7-integrin are present on blood NKT cells to varying levels, with few CLA^+^ NKT cells (6–19%), or CD62L^+^ NKT cells (11–24%), and a larger proportion expressing α4β7 (30–75%) (4, 87). However, the co-expression of specific adhesion molecules and chemokine receptors on NKT cells is required for homing into certain tissues. The frequency of NKT cells expressing both CD62L and CCR7 is much lower than the fraction of cells expressing either receptor alone (87). This is likely to explain the low frequency of NKT cells in peripheral lymph nodes. The identification of distinct NKT cell subsets that exhibit differential cytokine production and unique patterns of homing receptors suggests that different NKT cell subsets can be targeted to different tissues or sites of inflammation.

### Mouse NKT cells

Mouse NKT cells display significant differences in their chemokine receptor profiles and responsiveness to chemotactic ligands compared to human blood NKT cells. A majority of splenic mouse NKT cells express the receptors CCR9, CXCR3, CXCR4, and CXCR6 (88, 98), and TCRβ^+^NK1.1^+^ cells in Vα14 transgenic mice have elevated surface expression of CCR2, CCR5, and CCR9 (141). In contrast to human blood NKT cells, mouse blood, liver, spleen, and bone marrow NKT cells lack chemotactic responsiveness to ligands for CCR1, CCR2, CCR5, and CCR6 (88). Mouse NKT cells exhibited robust migration to the CXCR3 ligand monokine induced by gamma interferon (MIG; CXCL9) and the CXCR4 ligand stromal cell-derived factor 1 (SDF-1; CXCL12), while the CXCR6 ligand, CXCL16, only induced modest migration of NKT cells despite a large proportion of these cells expressing CXCR6 (Table 2) (88). This suggests that NKT cell responsiveness to CXCL16 and other chemokines is regulated. Consistent with this, responsiveness of CD8^+^ T cells to CXCL16 is dependent on activation (86), and increased chemotactic responsiveness to CXCL16 was observed in thymic NKT cells (91). Other tissue-specific differences among NKT cell subsets in terms of their chemokine receptor expression patterns or their chemotactic activity include the findings that a subset of NKT cells in the spleen, bone marrow, and blood, but not the liver, were responsive to the CCR7 ligand secondary lymphoid-tissue chemokine (SLC; CCL21), while a subset of CXCR5^+^ NKT cells were only present in the spleen and migrated in response to the CXCR5 ligand B cell-attracting chemokine 1 (BCA-1; CXCL13) (88). Ligands for CCR4 could mobilize lung NKT cells into the airways (108), but did not elicit chemotaxis of NKT cells derived from the spleen, liver, bone marrow, or blood (88), suggesting differences in CCR4 expression or regulation. Similarly, NKT cells in skin and peripheral lymph nodes express CCR6 and migrate in response to the ligand macrophage inflammatory protein 3α (MIP-3α; CCL20) (54), while spleen, liver, bone marrow, and blood NKT cells do not (88). As CCR6 expression on peripheral lymph node NKT cells correlates with a NKT-17 transcription profile, it will be interesting to correlate NKT cell localization and homing receptor expression in other tissues with the transcription factor expression patterns that have recently been used to classify NKT cell subsets.

**Table 2:** **Chemokine receptor expression and ligand responses on mouse NKT cells**.

Receptor	Expression (%)^a^	Chemotactic response^b^					Reference
		Spleen	Liver	Bone Marrow	Blood	Other	
CCR1	n.d.	−	−	−	−		(88)
CCR2	23	−	−	−	−		(88, 141)
CCR3	n.d.	−	−	−	−		(88)
CCR4	n.d.	−	−	−	−	Lung: ++	(88, 108)
CCR5	20–60	−	−	−	−		(88, 93, 141)
CCR6	PLN: 70–80	−	−	−	−	PLN: ++	(54, 88)
CCR7	Thymus: 15–60	+	−	+	+		(88)
CCR8	n.d.	−					(88)
CCR9	18–80	−					(88, 98, 141)
CCR10	n.d.	−					(88)
CXCR2	n.d.	−	−	−	−		(88)
CXCR3	80–96	++	++	++	++		(88)
CXCR4	55–58	+	+	++	++		(88)
CXCR5	Spleen: 35–38	+	−	−	−		(88)
CXCR6	92–94	+	n.d.	n.d.	n.d.	Thymus: +	(88, 91)
CX_3_CR1	n.d.	−	−	−	−		(88)
XCR1	n.d.	−	−	−	−		(88)

*^a^Range in reported frequency of receptor positive NKT cells*.

*^b^Net chemotactic migration: − (did not respond), + (<25%), and ++ (>25%)*.

*n.d., not determined; PLN, peripheral lymph nodes*.

Differential chemokine receptor expression on distinct NKT cell subsets suggests the potential to regulate homing to different tissue sites. However, the lack of functional chemotactic responses to many chemokine ligands suggests that chemokine receptor signaling is altered or regulated. Rather than contributing to tissue localization, some chemokines may play important roles in regulating NKT cell survival or effector functions. There is currently little known about the chemokine receptor profiles on activated NKT cells in comparison to resting NKT cells. *In vitro* activation of human NKT cells with α-GalCer upregulated CCR6 protein expression on DN NKT cells relative to CD4^+^ NKT cells, while the CD8^+^ NKT cell subset displayed increased mRNA for CX_3_CR1 (142). Whether these changes mediate alterations in NKT cell localization and/or activity remain to be seen.

## NKT Cell Homing and Maturation

During the developmental progression of thymocytes from immature DN precursors through the CD4^+^CD8^+^ double positive (DP) stage to mature conventional single positive cells, a subset of chemokine receptors regulate cellular trafficking and positioning through the cortex (CXCR4), subcapsular zone (CCR9), and medulla (CCR4, CCR7) (143–146). It is unclear whether positioning is similar during NKT cell development, as these cells are selected via DP thymocytes rather than thymic stromal cells (68, 147). However, while CCR4 is not expressed on thymic NKT cells, CCR7 controls NKT cell development by enabling access to IL-15 trans-presentation in the thymic medulla (148, 149). NKT cells upregulate the chemokine receptor CXCR6 during/after positive selection (91), which could also facilitate positioning within the medulla where the ligand CXCL16 is expressed (86).

**Figure 1:**
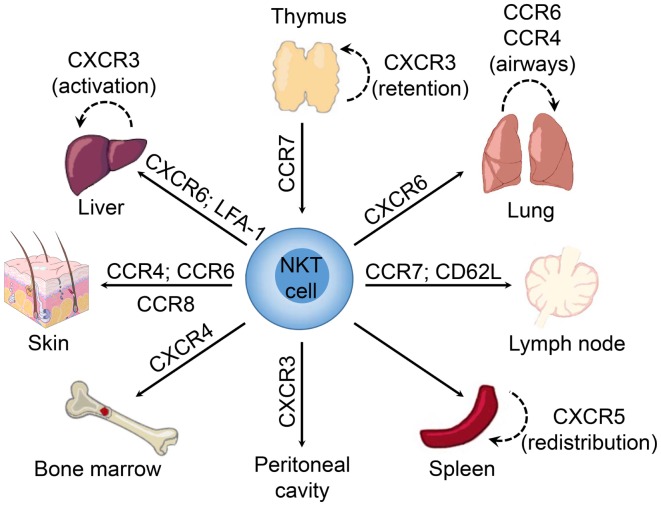
**Chemokine receptors involved in tissue-dependent NKT cell homing**. Following their development in the thymus, NKT cells emigrate to peripheral tissues (including liver, spleen, lung, bone marrow, lymph nodes, skin, and the peritoneum) where their accumulation and/or retention is regulated by adhesion molecules and chemokine–chemokine receptor interactions. Chemokine receptors and adhesion molecules associated with NKT cell redistribution within these tissues are indicated.

Interestingly, the NKT cell pool in the thymus contains both “immature” NK1.1^−^ and mature NK1.1^+^ subsets (150). The expression of T-bet during maturation of NK1.1^−^ NKT cells induces the expression of CCR5 and CXCR3 (74). The interaction of CXCR3 with interferon-γ-induced protein 10 (IP-10; CXCL10) expressed by medullary thymic epithelial cells retains mature NK1.1^+^ NKT cells in the thymus as a long-lived resident population (151) (Figure 1). However, it remains unclear what role these retained mature NKT cells might play within the thymus as they appear to be absent in humans (112), and conventional T cell development is unaffected in NKT-cell deficient (CD1d^−/−^) mice (152).

We have shown that NKT cells begin expressing high levels of CXCR6 in the thymus during the transition from CD4^+^CD8^+^ NKT cells to CD4^+^ and DN NKT cells following positive selection (91). CXCR6-deficiency does not affect thymic NKT cell development, but CXCR6^−/−^ mice exhibited a defect in the accumulation of mature CD1d-restricted NK1.1^+^ NKT cells in the periphery. Similarly, treatment of mice with a blocking antibody against CXCL16 did not inhibit accumulation of NK1.1^−^ recent thymic emigrants in the liver, but led to a defect in the accumulation of mature NK1.1^+^ NKT cells (91). These data point to a potential role for CXCR6 and CXCL16 in mediating maturation of NK1.1^−^ recent emigrant NKT cells, and retention and/or survival of mature NKT cells in the liver. A role for CXCR6 in retention is supported by the redistribution of NKT cells to the bone marrow in CXCR6^−/−^ mice (91), while others have also implicated CXCR6 in NKT cell survival (79, 90).

CXCR6^−/−^ mice also exhibited impaired cytokine production by liver and spleen NKT cells following activation with α-GalCer (2, 91). It is likely that CXCR6 delivers co-stimulatory signals to NKT cells as CXCL16 is expressed as a transmembrane protein on antigen-presenting cells (86, 153), and DCs from CXCL16^−/−^ mice are impaired in their ability to stimulate IFN-γ production from wild-type NKT cells (92). A reduction in IL-4 production by CXCR6^−/−^ NKT cells results from decreases in preformed IL-4 mRNA transcripts (91). Therefore, CXCR6 is critical for normal NKT cell development and function in addition to NKT cell homing and homeostasis. In addition to NKT cells, CXCR6 plays a role in regulating cytokine polarization in conventional T cell subsets. CXCR6 expression defines polarized subsets of Th1 and Th17 effector T cells *in vivo* (154, 155), and T cells from CXCR6^−/−^ mice exhibit impaired IFN-γ and IL-17 production in response to antigen restimulation *in vitro* (156).

Reporter mice in which the *Cxcr6* coding region was replaced with green fluorescent protein (*Cxcr6*^gfp/+^) have been used to show that liver NKT cells are localized within the vasculature, crawling along the luminal surface of liver sinusoids. Interestingly, although CXCR6-deficiency resulted in a significant reduction in NKT cell accumulation within the liver, it did not alter the crawling behavior of hepatic NKT cells (90), suggesting that other signals contribute to this behavior. The αLβ2-integrin (LFA-1) also appears to be important for the accumulation or retention of NKT cells within the liver, as mice deficient in LFA-1 have significantly reduced numbers of liver NKT cells (157, 158). Moreover, blockade of LFA-1 and intercellular adhesion molecule 1 (ICAM-1) interactions resulted in a marked reduction in hepatic NKT cell numbers with a concomitant increase in NKT cell frequency within the peripheral blood (110).

Similar to the liver, NKT cells accumulate in the lung via CXCR6 (91), and reside as an intravascular population (108, 110). This strategic positioning may facilitate sensing of airborne antigens or infection as airway exposure to glycolipids or microbial cell wall components induced accumulation of NKT cells in the lung interstitium and bronchoalveolar space (108). This rapid redistribution of NKT cells preceded local expansion of the intravascular cells and did not appear to involve recruitment of NKT cells from the periphery. Multiple chemokines were induced in the lung after exposure to airborne NKT cell ligands (108), including known NKT cell attractants such as thymus and activation regulated chemokine (TARC; CCL17), MIG/CXCL9, and BCA-1/CXCL13 (88, 89, 159). It was suggested that CCR4 may be important in regulating NKT cell redistribution in the lung, since aerosolized delivery of exogenous TARC/CCL17 was sufficient to drive extravasation of NKT cells into the lung parenchyma (108). These findings are consistent with previous work demonstrating that CCR4 mediates localization of NKT cells to the airways following aerosolized antigen challenge or delivery of α-GalCer to the lungs (160).

In contrast to circulating naïve conventional T lymphocytes, few NKT cells in mouse or human blood express both CD62L and CCR7 (87, 88), which is consistent with the relative scarcity of NKT cells within the lymph nodes. However, a subset of “immature” NK1.1^−^ NKT cells exhibited chemotaxis in response to CCR7 ligands *in vitro* (88). This was initially interpreted as a role for CCR7 in mediating the exit of immature NKT cells from the thymus, since chemotactic responsiveness to CCR7 ligands was not detected within mature NK1.1^+^ NKT cell subsets. However, this is also consistent with observations that a small subset of IL-17-generating NK1.1^−^ NKT cells accumulates preferentially within lymph nodes (3, 54). Within the lymph nodes, NKT cells are highly motile and are located mainly in the interfollicular region and in the medulla, but are absent in the paracortex where most naïve conventional T cells reside (161). In contrast to this *in situ* distribution under resting conditions, adoptively transferred NKT cells derived from the liver, spleen, or lymph nodes of TCR transgenic mice localized primarily to the lymph node paracortex (162). While the basis for these distinct distribution patterns was not investigated, it is possible that alterations in homing properties were induced by the manipulations involved in isolation, purification, and transfer of NKT cells.

Natural killer T cell populations resident in peripheral lymph nodes and skin exhibit an NK1.1^−^CD4^−^ phenotype, associated with expression of the retinoic acid receptor-related orphan receptor γτ (RORγτ) transcription factor, and generate IL-17 following activation (3, 54, 163, 164). Similar to Th17 cells, IL-17-producing NKT cells within the peripheral lymph nodes and skin in mice express CCR6 and migrate in response to the ligand MIP-3α/CCL20 (54), which has been shown to be involved in the recruitment of pathogenic Th17 cells to inflammatory sites in models of autoimmunity (165, 166). While it is unclear whether NKT-17 cells in the skin are distinct from those in the peripheral lymph nodes, it is thought that these cells are recruited via CCR6 and retained at epithelial sites by interactions between the αE-integrin (CD103) and E-cadherin (54). An NK1.1^−^ NKT cell population that produces IL-17 was also identified within the lung and shown to contribute to airway neutrophilia upon activation (56). While it is suggested that the NKT-17 lineage develops in the thymus of mice (55, 164), human and murine NKT cells can be differentiated into IL-17-producing cells in the presence of proinflammatory cytokines, such as IL-1β and IL-23, along with transforming growth factor-β (TGF-β) (167, 168). Furthermore, both CCR6^+^ and CCR6^−^ NKT cells from human blood contained cells that could produce IL-17 (167). This suggests plasticity in NKT cell populations, with the ability to be reprogramed in response to factors in the local tissue environment.

A subset of NKT cells in the spleen, but not in other tissues, expresses CXCR5 and actively migrates in response to BCA-1/CXCL13 (88), a chemokine that mediates homing to B cell zones in lymphoid tissues (169–172). Subsequent studies demonstrated that NKT_FH_ cells (CXCR5^+^PD-1^hi^) could provide cognate help to B cells, leading to the formation of antibody-producing plasma cells (65, 129, 173, 174). In contrast to the intravascular localization of NKT cells in the liver and lungs (90, 110), NKT cells in the spleen are widely distributed under basal conditions, dispersed throughout the red and white pulp (175), the periarteriolar lymphoid sheath (110), the marginal zone (176), and occasionally in close proximity to the vasculature (177). Exogenous glycolipid antigens or infection with *Streptococcus pneumoniae* induced rapid accumulation and immobilization of splenic NKT cells in close proximity to marginal zone DCs and macrophages (175, 176). Importantly, the number of splenic CD1d-tetramer^+^ NKT cells were not significantly altered, suggesting that the accumulation in these areas of the spleen is due to redistribution and not enhanced recruitment of NKT cells from the peripheral blood (175).

## Role of NKT Cells in Microbial Host Defense

Many studies have implicated roles for NKT cells in the immune responses elicited by microbial pathogens (6, 178–180). NKT cells respond to a range of infectious organisms through the recognition of microbial lipids presented via CD1d^+^ antigen presenting cells (181–187). For example, α-galactosyldiacylglycerol from *Borrelia burgdorferi* (the causative agent of Lyme disease) (185), lipophosphoglycan from *Leishmania donovani* (188), α-glucosyldiacylglycerol from *S. pneumoniae* (189), α-glucuronosylceramide and α-galacturonosylceramide from *Sphingomonas* species (182–185), and a cholesteryl α-glucoside from *Helicobacter pylori* (186) are all recognized by the invariant TCR of NKT cells within the context of CD1d.

However, NKT cell activation is not restricted to microbes that contain lipid antigens recognized directly by the Vα14–Jα18 TCR on *i*NKT cells. Other microbial products stimulate antigen presenting cells via pattern recognition receptors (TLRs, NOD-like receptors, etc.), causing enhanced accumulation of weak self-lipid antigens and the production of NKT cell-stimulating cytokines (IL-12, IL-18, and type I IFNs) (46, 184, 190–193). Endogenous lipid ligands induce weak signaling through the NKT cell TCR that is not sufficient for full NKT cell activation, but primes NKT cells to produce IFN-γ upon exposure to the cytokines IL-12 and IL-18 (194). Moreover, there is evidence that CD1d-presented antigens may not be required and IL-12, IL-18, or type I IFNs alone or in combination may be sufficient to drive NKT cell activation and IFN-γ production (193, 195).

The mode of activation may have implications for NKT cell localization and effector functions. *In vitro*, NKT cells form stable conjugates with α-GalCer-pulsed DCs and subsequently lose motility, whereas NKT cells incubated with DCs in the presence of exogenous IL-12 and IL-18, or LPS-treated DCs, exhibit unaltered migration patterns (194). Consistent with this, NKT cell activation through intravenous delivery of exogenous glycolipid caused CD1d-dependent NKT cell arrest within liver sinusoids (90, 196) and induced rapid accumulation of NKT cells in the marginal zone of the spleen (175, 176). In contrast, while IL-12 and IL-18 treatment induced CD1d-independent arrest in liver sinusoids (196), these cytokines did not induce NKT cell redistribution to the marginal zone within the spleen (175). This suggests a role for CD1d engagement and cell–cell interactions in regulating the specific localization and redistribution of NKT cells, while cytokine stimulated NKT cells likely adhere to local integrin ligands in response to inside out signaling. There could also be differences in the chemotactic signals and localization gradients elicited by antigenic versus cytokine stimuli.

## NKT Cells in Bacterial Infections

### *Borrelia* *burgdorferi*

Lyme disease is caused by *B. burgdorferi*, a bacterial spirochete that generates the NKT cell-stimulating glycolipid, α-galactosyldiacylglycerol (185). NKT cell-deficient mice (CD1d^−/−^ and Jα18^−/−^) exhibit increased bacterial burden when infected with *B. burgdorferi* (197, 198). Under homeostatic conditions, NKT cells actively crawl within hepatic sinusoids (90). However, in mice systemically infected with *B. burgdorferi*, the majority (~80%) of sinusoidal NKT cells arrested and formed clusters in stable contact with *B. burgdorferi*-containing Kupffer cells (197). Interestingly, antibody blockade of either CXCR3 or CD1d inhibited NKT cell arrest and cluster formation (197). Kupffer cells release substantial amounts of MIG/CXCL9 early following infection with *B. burgdorferi* (197), suggesting that a chemotactic gradient facilitates recruitment and interaction of CXCR3^+^ NKT cells with CD1d^+^ Kupffer cells. This response reflected a redistribution of hepatic NKT cells as there was little recruitment of additional NKT cells to the liver.

Intriguingly, the most prominent phenotype in *B. burgdorferi*-infected NKT cell-deficient mice was a greater abundance of bacteria in the joints (197, 198). This suggests that NKT cells play a role in limiting the emigration of *B. burgdorferi* out of the vasculature in Lyme disease-associated arthritis. In contrast to the intravascular distribution and patrolling behavior of NKT cells in the liver, Lee et al. (199) found that NKT cells in the joint were distributed throughout the extravascular tissue with the majority remaining stationary and in close contact with the blood vessels. Following *B. burgdorferi* infection, the spirochetes were found to adhere to the inner wall of joint blood vessels and attempt to extravasate into the tissue. Extravascular NKT cells in the vicinity of the adherent pathogen increased their crawling activity, suggesting the release and recognition of pathogen- or host-derived chemotactic factors, possibly complement-derived anaphylatoxins (199). Moreover, NKT cells played a critical role in clearance of *B. burgdorferi* from the joint tissue via direct granzyme-dependent killing (199). In contrast to the CD1d-dependent responses to *B. burgdorferi* in the liver, NKT cell-mediated pathogen recognition and killing activity in the joint was not dependent on CD1d interactions. This study highlights the importance of NKT cell positioning with respect to their anti-microbial function and identifies a functionally unique subset of bactericidal NKT cells in the joint, since liver and spleen NKT cells were unable to directly recognize and kill *B. burgdorferi*. Interestingly, NKT cells were found to be present in the normal joint under homeostatic conditions (199), and infection with *B. burgdorferi* did not result in enhanced accumulation of NKT cells within the joints in mice (198).

Extravascular NKT cells have also been detected in knee joints of *B. burgdorferi* infected patients (199, 200). Although there are fewer NKT cells in human joints compared to mice, synovial fluid from patients with osteoarthritis, rheumatoid arthritis, and Lyme arthritis contained increased numbers of activated NKT cells (199, 200). It is unclear if this represents redistribution or expansion of local NKT cells or recruitment of NKT cells from other sites. While this appears to contrast with the lack of increased NKT cell accumulation in mice with Lyme borreliosis, it could result from differences in the time course of disease or differences in the precise compartment within the joint from which samples were collected. Lee et al. (199) demonstrated that NKT cells were not uniformly distributed throughout the joint but rather were found predominantly at the joint surface, outside of the joint capsule. The increased number of NKT cells detected in synovial fluid samples from arthritis patients could represent a redistribution of extravascular joint-resident NKT cells into the joint capsule, which may be promoted by the presence of *B. burgdorferi* within synovial fluid. Consistent with this, Katchar et al. (200) observed a significantly higher proportion of NKT cells in the synovial fluid of patients with antibiotic-responsive Lyme arthritis (*B. burgdorferi* present in joint fluids in all patient samples) compared to the nearly undetectable levels of NKT cells in those with antibiotic-refractory Lyme arthritis (10 of 15 patients lacked detectable *B. burgdorferi* in joint fluids). T cells and NK cells were detected at similar levels in the synovial fluid of both patient groups, further suggesting that NKT cells play a key role in host defense against *B. burgdorferi*, and their absence may contribute to excessive inflammation and immune dysregulation in the joints of antibiotic-refractory Lyme arthritis patients.

### *Chlamydia* 

*Chlamydia* species are obligate intracellular pathogens that can cause numerous disease states in humans, including lung infection (201), gastrointestinal infection (202), urogenital infection (203), and reactive arthritis (204). Using an animal model of *C. trachomatis*-induced arthritis, Bharhani et al. (205) demonstrated that NKT cells play a role in ameliorating joint inflammation. Mice deficient in NKT cells (CD1d^−/−^) exhibited enhanced arthritis severity, while α-GalCer treatment of *C. trachomatis*-infected wild-type mice increased the accumulation of NKT cells within synovial tissues, reduced bacterial load, suppressed expression of inflammatory chemokines [macrophage inflammatory protein-2 (MIP-2) and IP-10/CXCL10], and decreased infiltration of inflammatory cells into the inflamed joint. Moreover, while Bharhani et al. (205) could not detect NKT cells in synovial tissues of control mice, synovial NKT cell populations were detected in *C. trachomatis*-infected mice, suggesting active recruitment of NKT cells to inflamed joints in these mice. However, since others have identified NKT cells in the joints of control mice (199), it is unclear whether the increased proportion of synovial NKT cells following α-GalCer treatment of *C. trachomatis*-infected mice was due to further recruitment of NKT cells, or resulted from local NKT cell expansion.

Natural killer T cells have been shown to respond rapidly to infection and regulate microbial immunity in response to *C. muridarum* infections in the lung and genital tract of mice (206, 207), where treatment with α-GalCer enhances IFN-γ production to increase host resistance (207). Jiang et al. (208) reported elevations in bacterial burden and inflammatory cell infiltrate in the genital tract of CXCR5^−/−^ mice infected with *C. muridarum*. While CXCR5-deficiency did not alter NKT cell accumulation in the genital tract, CXCR5^−/−^ mice exhibited increased NKT cell activation *in vitro* and *in vivo* in response to *C. muridarum* infection. Enhanced production of IFN-γ by NKT cells from CXCR5^−/−^ mice suggests a possible role for CXCR5 in regulating the activity of NKT cells. However, enhanced NKT cell activity in CXCR5^−/−^ mice did not provide greater protection against *C. muridarum* genital tract infection *in vivo* (208), implicating important roles for other CXCR5^+^ immune cells in mediating protective responses.

### *Streptococci/Cryptococci* 

CD1d-dependent activation of NKT cells in response to α-glucosyldiacylglycerol has been demonstrated in mice infected with *S. pneumoniae* and group B *Streptococcus* (causative agents of neonatal infections in humans) (189). This supports earlier studies implicating a critical role for NKT cells in early host defense against *S. pneumoniae* infection via their production of IFN-γ and recruitment of neutrophils to infected lungs (25, 209). Consistent with a TCR-dependent activation mode, NKT cell activation and cytokine production in response to *S. pneumoniae* was associated with increased NKT cell GFP expression in Nur77-GFP mice, a reporter strain that upregulates GFP in response to TCR-mediated stimuli, but not TCR-independent inflammatory stimuli (210, 211). The frequency of lung NKT cells increased following *S. pneumoniae* infection, and following intratracheal infection with the fungal pathogen *Cryptococcus neoformans* (25, 212). In both cases, the increased NKT cell frequency may be dependent upon CCR2 and monocyte chemotactic protein 1 (MCP-1)/CCL2 mediated recruitment since the frequency of NKT cells was significantly reduced in the lungs of infected CCL2^−/−^ mice compared to wild-type mice (25, 212). However, further studies are required to elucidate the relative contributions of NKT cell expansion and mobilization from lung parenchyma versus the recruitment of circulating NKT cells from the blood.

Natural killer T cells were found to promote antibody isotype switch, affinity maturation, and long-term memory B cell responses against pneumococcal capsular polysaccharides following delivery of a liposome nanoparticle vaccine containing *S. pneumoniae* capsular polysaccharide and a NKT cell-stimulating lipid (213). Antibody responses elicited by the vaccine were dependent upon cognate CD1d-restricted interactions between NKT cells and B cells, a process that might be predicted to require direct B cell help provided by NKT_FH_ cells. However, very little induction of CXCR5^+^PD-1^hi^ NKT_FH_ cells was observed in immunized mice, suggesting a mostly extrafollicular response. These findings suggest that the inclusion of NKT cell ligands in microbial antigen-presenting liposomal particles may represent a simple and effective alternative to the conjugate vaccines currently used to elicit strong cognate help to B cells to promote protective and long lasting antibody responses.

### Bacterial sepsis

Chemokine receptor-mediated regulation of lymphocyte activation and homing has also been described during sepsis. In a mouse model of sepsis caused by cecal ligation and puncture (CLP), Herzig et al. (214) observed a CXCR3-dependent increase in peritoneal NK cell and T cell accumulation, likely due to increased concentrations of the chemokines MIG/CXCL9 and IP-10/CXCL10 in the peritoneal cavity. In contrast, CLP did not result in an increased accumulation of NKT cells within the peritoneal cavity, but did decrease CXCR3 expression on NKT cells in the liver. The authors suggested this could be due to NKT cells becoming activated during CLP, causing the internalization and down-regulation of CXCR3 (214). Interestingly, the peritoneal cavity of CXCR3^−/−^ mice was nearly devoid of NKT cells prior to and following induction of CLP, while CXCR3-deficiency had no impact on the numbers of NKT cells in the spleen (214). This suggests CXCR3 is important for the accumulation of NKT cells within the peritoneum under normal physiological conditions.

The anaphylatoxins (C3a and C5a), generated during complement activation, are chemotactic molecules that may also influence NKT cell localization and activation. NKT cells express high levels of mRNA, but not protein, for C5a receptor (C5aR) under homeostatic conditions (215). However, upon *Escherichia coli*-induced sepsis in mice, C5aR protein is rapidly expressed on splenic NKT cells (215). Interestingly, NKT cells from C5aR^−/−^ mice infected with *E. coli* expressed lower levels of the activation marker CD69 and had reduced secretion of IFN-γ and TNF, suggesting that C5aR signaling regulates the activation of NKT cells in this model (215). Cognate C5a/C5aR interactions on NKT cells were also identified as a critical factor for NKT cell recruitment during sepsis based on the observations that C5aR^−/−^ mice had markedly reduced numbers of NKT cells in the spleen and peritoneal cavity following infection (215). Furthermore, *E. coli* infection induced greater accumulation of C5aR^+^ versus C5aR^−^ NKT cells in the spleen of mixed bone marrow chimeras (215). The absence of C5aR and NKT cells were both associated with increased survival following infection, suggesting NKT cells contribute to the overwhelming inflammation in sepsis.

### *Mycobacterium* 

Activation of NKT cells via α-GalCer has been shown to contribute to protection against *M. tuberculosis* in mice (216). Moreover, adoptive transfer of NKT cells decreased mycobacterial burden in the lung and spleen, and NKT cells were able to inhibit intracellular replication of *M. tuberculosis* within infected macrophages *in vitro* (217). CXCR6 on lung T cells has been proposed as a marker for protective immunity to *M. tuberculosis* after intranasal immunization in mice, with CXCR6 and CXCL16 playing a critical role in mediating the localization of T cells within the airways (218). It is possible that the CXCR6–CXCL16 axis is also important for NKT cell localization in these tissues as well, since lung NKT cells are reduced under baseline conditions in CXCR6^−/−^ mice (91).

Phosphatidylinositol-mannosides (PIMs) are phospholipid antigens located in the membranes of mycobacteria, some of which activate human and murine NKT cells via CD1d (181). Despite its inability to trigger expansion of NKT cells (181), PIM_2_ causes recruitment of NKT cells to the skin upon subcutaneous injection (219). Although this recruitment is TCR-independent, the mechanism is unclear. Intratracheal infection of mice with *M. bovis* bacillus Calmette-Guérin (BCG) induced NKT cell mobilization into the airways, which was profoundly impaired in CCR6^−/−^ mice (220). In this study, lung parenchymal NKT cells in *M. bovis* BCG-infected wild-type mice were found to have high expression of CCR6, likely imbuing them with responsiveness to the high levels of MIP-3α/CCL20 induced in the lungs by *M. bovis* BCG infection (220). The number of NKT cells in the lung parenchyma did not differ between wild-type and CCR6^−/−^ mice, suggesting that the >90% reduction in mobilization of NKT cells to the luminal airways in *M. bovis* BCG-infected CCR6^−/−^ mice was due to a requirement for CCR6 in the airway infiltration but not lung localization of NKT cells (220).

## NKT Cells in Viral Infections

### Hepatitis viruses

In patients with chronic hepatitis B virus (HBV) infections, the frequency of circulating CD4^−^ NKT cells is lower than that in asymptomatic carriers or healthy controls, but infection did not impair the ability of peripheral NKT cells to produce IFN-γ or IL-4 in response to activation with either α-GalCer or the mitogen phorbol 12-myristate 13-acetate (PMA) (221). Interestingly, the frequency of NKT cells increased significantly following antiviral therapy (221). However, it is unclear whether this reflects changes in proliferation, survival, or homing of NKT cells.

Although there has not been an exhaustive examination of chemokine receptors on NKT cells in patients infected with HBV, the frequency of NKT cells expressing CCR5 and CCR6 was comparable between chronic HBV patients and healthy controls (221). The migratory responses of NKT cells from chronic HBV patients to CCR5 and CCR6 ligands were either very modest (RANTES/CCL5) or not detectable (MIP-3α/CCL20) compared to medium alone (221). However, the responses in this study were not compared to chemotactic responses of NKT cells from healthy controls.

A wide spectrum of clinical disease can occur following HBV infection, ranging from an asymptomatic carrier state, to self-limiting acute disease, chronic hepatitis, cirrhosis, liver failure, and hepatocellular carcinoma (222). A study in India reported a significantly increased frequency of circulating NKT cells (CD3^+^CD56^+^CD16^+^) and higher levels of MIP-1β/CCL4 among patients with acute HBV infection, but not HBV-induced liver failure, compared to healthy controls (223). However, a separate study reported a decline in circulating NKT cell (CD3^+^CD56^+^) frequencies in acute HBV patients in the first few weeks following hospital admission, which the authors suggested could be due to trafficking of NKT cells to the liver where they play a role in local HBV immunity (224). These studies need to be interpreted cautiously as CD3^+^CD56^+^/CD16^+^ populations exhibit only partial overlap with the *i*NKT cell population.

Interestingly, Inoue et al. (225) reported higher surface expression (mean fluorescence intensity) of CXCR3 on circulating NKT cells isolated from patients with chronic hepatitis C virus (HCV) infection, while expression of CCR4, CCR7, or CD62L did not differ compared to healthy donors. The enhanced expression of CXCR3 may facilitate the trafficking of NKT cells to or within the liver due to the increased hepatic levels of MIG/CXCL9 and IP-10/CXCL10 during HCV infection (226, 227). Whether increased numbers of hepatic NKT cells during chronic HCV infection would be beneficial is unclear since NKT cells from HCV^+^ patients produce more IL-13 and other Th2 cytokines (225), which could contribute to liver fibrosis during chronic viral hepatitis (228).

### Dengue virus

A recent study examining the role of NKT cells in the pathogenesis of dengue virus infection in humans found that peripheral NKT cell numbers were not altered over the course of dengue virus infection (229). However, NKT cells displayed an activated phenotype that correlated with increased disease severity (229). Similarly, NKT cells exhibit an activated phenotype and appear to play a detrimental role during dengue infection in mice (230). NKT cell-deficient mice (Jα18^−/−^) exhibited resistance to lethal infection, which was associated with decreased systemic and local inflammatory responses, reduced production of inflammatory cytokines (IL-6, IFN-γ, and IL-12p40), and reduced levels of CXCL1, a chemokine known to rapidly mobilize and activate neutrophils (230). In wild-type mice, mast cells responding to dengue virus infection upregulated chemokine expression (RANTES/CCL5, SDF-1/CXCL12, and fractalkine/CX_3_CL1), and mediated recruitment of NKT cells (CD3^+^NK1.1^+^) into the skin at sites of dengue virus infection (231). Taken together, these data suggest NKT cells play a critical role in the pathogenesis of dengue disease.

### Influenza virus

Influenza virus is a respiratory pathogen that can be the cause of serious airway disease, particularly among children and the elderly. The number of circulating NKT cells were reduced in patients with severe cases of pandemic H1N1 influenza infection (232), but it is unclear whether this impacted disease progression. In rodent models, NKT cells play protective roles in influenza infection through multiple mechanisms. They have been reported to suppress excessive monocytic infiltrate (233), influence the generation of virus-specific CD8^+^ T cell responses (234), enhance the cytolytic activities of NK cells and virus-specific CD8^+^ T cells (via IFN-γ production) (235), and selectively lyse virally infected cells through a CD1d-dependent mechanism (233). Interestingly, NKT cells were also found to reduce the expansion and immunosuppressive activity of influenza-induced myeloid-derived suppressor cells (236), an immune modulatory activity of NKT cells that has also been shown in cancer models (237).

Much of the research on NKT cells in influenza infection focuses on the potential for NKT cell-stimulating glycolipid agonists such as α-GalCer to act as vaccine adjuvants. Several studies have shown that intranasal immunization of inactivated influenza or a live attenuated influenza vaccine, together with α-GalCer or its derivatives, induced high levels of influenza-specific systemic IgG and mucosal IgA, influenza-specific CD8^+^ T cell memory responses, and complete protection against influenza viral challenge in mice (238–242). Intranasal administration of α-GalCer was shown to increase the NKT cell populations in nasopharyngeal-associated lymphoid tissue (NALT) and regional cervical lymph nodes, but not the spleen, indicating that nasal administration of α-GalCer influences the local NKT cell population size without altering the systemic NKT cell population (242). Interestingly, expression of CXCL16 was upregulated in NALT and cervical lymph nodes following vaccination. NKT cell accumulation within these tissues and influenza-specific mucosal IgA levels were reduced in CXCL16^−/−^ mice (242). Therefore CXCR6–CXCL16 interactions contribute to the increased population of NKT cells following nasal influenza vaccination either by regulating homing or expansion of these cells. It would be interesting to determine whether influenza vaccination in conjunction with intranasal α-GalCer administration also increases the NKT cell population in the lung and/or airways, and if so, whether this increase is also impaired in CXCL16^−/−^ mice.

### Human immunodeficiency virus

Natural killer T cells are highly susceptible to infection with HIV-1 due to the expression of multiple co-receptors for viral fusion and entry, including CD4 and the chemokine receptors CCR5, CXCR4, and CXCR6 (138, 243–248). Indeed, NKT cell frequency is reduced in patients with HIV-1 infection, with a preferential depletion of the CD4^+^ NKT cell subset prior to depletion of conventional CD4^+^ T cells (243, 248–250). Interestingly, the frequency of CCR5^+^ NKT cells was higher in HIV-1^+^ patients, while CXCR6^+^, CCR2^+^, and CCR7^+^ NKT cell frequencies were reduced compared to healthy individuals (251, 252). Circulating NKT cell populations have been shown to recover early following effective antiretroviral therapy, but treatment failed to restore CXCR6 or CCR2 expression on NKT cells (252). Some have speculated that the rapid recovery of NKT cells after treatment is partly due to NKT cell redistribution from tissue sites to the circulation, a phenomenon that has been observed for conventional T cells in HIV-1^+^ patients following therapy (253, 254).

In addition to influencing circulating NKT cell numbers, HIV-1 infection also impairs the proliferative and cytokine-producing capacities of persisting NKT cells in chronic HIV-1^+^ patients (255, 256). However, the role of NKT cells in HIV-1 infection remains unclear since some studies report no correlation between NKT cell numbers and HIV disease progression (249), while others have suggested an association between higher levels of CD4^+^ NKT cells and lower plasma viremia (243). NKT cell activation with α-GalCer has shown promise as a vaccine adjuvant in animal models when combined with delivery of HIV-1 DNA and peptide antigens (257, 258), suggesting that NKT cells have the potential to play important roles during HIV-1 infection.

Natural killer T cells from HIV-1^+^ patients expand *in vitro* following treatment with IL-15 and IL-12 (251), and a combination of antiretroviral therapy with exogenous IL-2 promotes a greater increase in circulating NKT cell numbers than standard therapy alone (259). Therefore, the reduced peripheral NKT cell population in HIV-1-infected individuals is likely due to a combination of factors, which include direct HIV-1 infection of NKT cells and subsequent cell death, tissue redistribution of NKT cells, and impaired generation and/or responsiveness to cytokines that promote NKT cell survival. A better understanding of the mechanisms contributing to NKT cell depletion in HIV-1^+^ patients could lead to the development of new therapeutic strategies to restore NKT cell numbers and lead to better clinical outcomes following HIV-1 infection.

## Conclusion and Outstanding Questions

Our understanding of the distinct phenotypic and functional subsets of NKT cells continues to improve, allowing for clearer interpretations of how NKT cells contribute to health and disease. Under homeostatic conditions, NKT cells can be found in many tissues throughout the body, and NKT cell accumulation within specific sites can be linked to the expression of specific chemokine receptors and adhesion molecules that mediate tissue homing, retention, and/or survival (e.g., liver accumulation via CXCR6 and LFA-1). Upon activation, local NKT cell populations can expand and use chemotactic signals to relocalize within a tissue. However, in most cases, there is little or no evidence that NKT cells are recruited to sites of inflammation from the blood or other tissues. Despite their low numbers, NKT cells influence the magnitude and polarization of immune responses in a wide array of contexts ranging from antimicrobial and antitumor responses to autoimmunity. However, many questions remain regarding the roles of NKT cells in these conditions.

Patients with chronic microbial infection, autoimmune disorders, and malignancies often have alterations in the number and functional activity of NKT cells (7, 119, 122, 260, 261). Some have speculated that reduced NKT cell numbers in the peripheral blood of these patients are linked to NKT cell trafficking to diseased tissue sites associated with these disorders (120, 224, 262–264). However, reduced NKT cell numbers or other NKT cell defects in many disease states may be associated with the standard therapies used to treat the disease rather than the disease itself. For example, reduced NKT cell frequencies were not observed in patients with myelodysplastic syndrome or multiple myeloma prior to treatment, but defects in peripheral NKT cells emerged following initiation of standard therapy (265, 266). In most diseases in which NKT cell numbers are affected, further investigation is required to track whether alterations in NKT cell numbers are due to altered trafficking or redistribution of NKT cells to various tissue sites. This will require that studies examine patients multiple times over the course of disease development and ideally include multiple tissues and treatment-naïve groups, rather than only examining patients on a single occasion after disease onset as most studies have done to date. Doing so will allow clearer correlations to be made between altered NKT cell numbers/function (i.e., altered subset frequencies and cytokine production) and disease progression, and ultimately provide evidence as to whether NKT cell defects are a cause or consequence of the disease process.

Under homeostatic conditions, NKT cells appear to be tissue-resident populations and exhibit very little exchange with NKT cells in the circulation, as evidenced by studies using parabiotic congenic mice. NKT cells in the blood in these pairs reach almost equal (50%) chimerism, while those in the lung, liver, spleen, lymph nodes, bone marrow, and other tissues did not recirculate, with nearly all NKT cells in these tissues originating from the host (59, 108, 110). In contrast, conventional CD4^+^ and CD8^+^ T cells, B cells, and NK cells rapidly recirculate and equilibrated in these tissues (110). This poses interesting questions regarding NKT cell redistribution during microbial infection. Multiple studies described above have observed greater NKT cell accumulation in affected tissues in a variety of infections. However, in many studies, the authors have not distinguished between the possibilities of NKT cell recruitment into the tissue versus expansion and relocalization of tissue resident NKT cells. Regardless, their accumulation at sites of infection ensures NKT cells are exposed to potential activating stimuli, either directly through specific recognition of microbial lipid antigens or indirectly through self-glycolipid and cytokine stimulation. Intriguingly, NKT cells may not need to be present in an affected tissue site in order to respond and subsequently influence the immune response within the host. For example, liver NKT cells responding directly to noradrenergic neurotransmitters were shown to release anti-inflammatory cytokines that induced a state of immune suppression that rendered mice susceptible to bacterial infection following ischemic cerebral stroke (47). Similarly, NKT cells are activated in the liver during the induction of contact hypersensitivity reactions (267). Therefore, a lack of NKT cell accumulation within inflamed peripheral tissues during infection may not preclude effective (or deleterious) antimicrobial immune responses mediated by tissue-resident NKT cells at a distant site.

A number of NKT cell subsets have been described that exhibit distinct phenotypes and functions in terms of surface marker expression and cytokine profiles. For example, lymph node resident CCR6^+^CD4^−^NK1.1^−^NKT cells described earlier express the transcription factor RORγτ and produce IL-17 in response to inflammatory signals (54). Unique transcriptional programs have been identified for NKT-1, NKT-2, NKT-10, and NKT-17 subsets of NKT cells within the thymus (52–58). Recent data reveal that NKT cell lineage fate is regulated by lethal-7 (let-7) microRNAs (miRNAs), which target *Zbtb16* mRNA (encoding PLZF) to post-transcriptionally regulate the expression of PLZF protein (268). The expression of let-7 miRNAs was dynamically regulated during NKT cell development, with IL-15 and other stimuli present in the thymic medulla contributing to upregulated let-7 miRNAs and reduced levels of PLZF protein during NKT cell differentiation. NKT cells with downregulated levels of PLZF differentiated into IFN-γ-producing NKT-1 cells. Conversely, reduced expression of let-7 miRNAs resulted in greater levels of PLZF protein and a thymic bias toward NKT-2 and NKT-17 differentiation. However, this bias was less evident in the peripheral tissues (liver, spleen, and lymph nodes) of mice with reduced let-7 miRNAs (268), suggesting the relative frequencies of NKT cell effector subsets are influenced by differential migration and expansion of certain NKT cell effector lineages within specific tissue microenvironments. Nevertheless, it will be important to determine whether different NKT cell subsets *in vivo* represent committed lineages of cells with distinct homing receptors or if these subsets exhibit plasticity and are able to adopt various functional roles depending upon soluble and cell-associated signals received within a given tissue microenvironment. Furthermore, a focused research effort is needed to investigate the relative roles of distinct NKT cell subsets during microbial infection.

## Conflict of Interest Statement

The authors declare that the research was conducted in the absence of any commercial or financial relationships that could be construed as a potential conflict of interest.
